# Non-invasive methods for the determination of body and carcass composition in livestock: dual-energy X-ray absorptiometry, computed tomography, magnetic resonance imaging and ultrasound: invited review

**DOI:** 10.1017/S1751731115000336

**Published:** 2015-03-06

**Authors:** A. M. Scholz, L. Bünger, J. Kongsro, U. Baulain, A. D. Mitchell

**Affiliations:** 1 Livestock Center Oberschleißheim, Ludwig-Maximilians-University Munich, Sankt-Hubertusstrasse 12, 85764 Oberschleißheim, Germany; 2 SRUC, Animal and Veterinary Sciences, Roslin Institute Building, Easter Bush, Midlothian, Scotland EH25 9RG, UK; 3 Norsvin, Department of Animal and Aquacultural Sciences, c/o Norwegian University of Life Sciences, PO Box 5003, N-1432 Ås, Norway; 4 Institute of Farm Animal Genetics, Friedrich-Loeffler-Institut, Hoeltystr.10, 31535 Neustadt, Germany; 5 Agricultural Research Service (Retired), US Department of Agriculture, 10300 Baltimore Avenue, BARC-West, Beltsville, MD 20705, USA

**Keywords:** animal, body composition, X-ray attenuation, magnetic resonance imaging, ultrasound

## Abstract

The ability to accurately measure body or carcass composition is important for performance testing, grading and finally selection or payment of meat-producing animals. Advances especially in non-invasive techniques are mainly based on the development of electronic and computer-driven methods in order to provide objective phenotypic data. The preference for a specific technique depends on the target animal species or carcass, combined with technical and practical aspects such as accuracy, reliability, cost, portability, speed, ease of use, safety and for *in vivo* measurements the need for fixation or sedation. The techniques rely on specific device-driven signals, which interact with tissues in the body or carcass at the atomic or molecular level, resulting in secondary or attenuated signals detected by the instruments and analyzed quantitatively. The electromagnetic signal produced by the instrument may originate from mechanical energy such as sound waves (ultrasound – US), ‘photon’ radiation (X-ray-computed tomography – CT, dual-energy X-ray absorptiometry – DXA) or radio frequency waves (magnetic resonance imaging – MRI). The signals detected by the corresponding instruments are processed to measure, for example, tissue depths, areas, volumes or distributions of fat, muscle (water, protein) and partly bone or bone mineral. Among the above techniques, CT is the most accurate one followed by MRI and DXA, whereas US can be used for all sizes of farm animal species even under field conditions. CT, MRI and US can provide volume data, whereas only DXA delivers immediate whole-body composition results without (2D) image manipulation. A combination of simple US and more expensive CT, MRI or DXA might be applied for farm animal selection programs in a stepwise approach.

## Implications

The ability to accurately and precisely measure body or carcass composition is important for performance testing, grading and finally the selection or payment of meat-producing animals. Advances especially in non-invasive techniques are mainly based on the development of electronic and computer-driven methods in order to provide objective phenotypic data. This review provides a summary of the recent developments in the application of dual-energy X-ray absorptiometry, X-ray computed tomography, magnetic resonance imaging and ultrasound.

## Introduction

Although meat consumption in Europe and worldwide is not increasing at the same rate, meat from farm animals will continue to be the major source of protein for human nutrition throughout the world (OECD/Food and Agriculture Organization of the United Nations, 2014). In order to provide a fair and comparable payment for farmers, it is necessary to base the classification of carcasses or meat-producing animals on harmonized procedures with the least impact on the quality and quantity of the products for human consumption. Non-invasive grading or classification procedures have preference over invasive procedures like dissection or chemical analysis. The ability to accurately and precisely measure body composition or carcass composition is also important for applications related to *PHENOTYPING* in performance testing and breeding programs or scientific studies focusing on growth, nutrition, genetics, housing and behavior or farm animal well-being.

## Background

Humans started to classify or select animals with the domestication of wild animals thousands of years ago. This classification and selection process was (and still is) mainly based on visual and tactile appraisal with a preference for less-aggressive and easily fed animals. Apart from selection procedures based on the form, size, weight, speed, behavior, fertility, antlers, horns or coat color, it took thousands of years until ‘more’ scientifically based procedures for farm animal evaluation or classification were invented. In almost all cases, farm or wild animals had or have to be sacrificed in order to be able to process the products for human consumption or utilization – with the exception of milk, egg, wool, work, company or manure. Tissue dissection, however, is still the main European reference standard for the approval of carcass classification procedures or formulas (Nissen *et al.*, 2006^1^).

First ‘non-invasive imaging’ methods on farm animals were tested by Kronacher and Hogreve (1936) and Hogreve (1938) using X-radiography in order to study the pelvis shape of different pig breeds and the adipose tissue deposition of fattening pigs, respectively. The first studies using the specific velocity of ultrasound (US; >20 kHz) in different body tissues were started in meat-producing farm animals by Temple *et al.* (1956). More than 30 years ago, the first papers dealing with X-ray-based computed tomography (CT; Skjervold *et al.*, 1981) and nuclear magnetic resonance imaging (tomography – MRI: Groeneveld *et al.*, 1983; even earlier spectroscopy, Casey and Miles, 1974) for the evaluation of meat or carcass and body composition of farm animals were published. Early attempts to cope automatically with MRI inhomogeneity were made by Scholz *et al.* (1993) using a cluster analysis for the segmentation into fat and muscle tissue of pigs *in vivo* only after defining a region of interest of the body part MR scanned. The first dual-energy X-ray absorptiometry (DXA) studies – especially regarding farm animal body composition – started with Mitchell *et al.* (1996).

Since then, technical progress continued providing ‘bigger, quicker and smarter’ non-invasive imaging or scanning devices for the determination of body and/or carcass composition measurements in farm animal selection programs. Besides Australia, New Zealand, Norway and the United Kingdom, quite a few countries like, for example, Austria, Canada, Denmark, France, Germany, Hungary, Ireland, Spain, Sweden and the United States of America use(d) CT (e.g. Junkuszew and Ringdorfer, 2005; Romvari *et al.,* 2006; Font-i -Furnols and Gispert, 2009; Vester-Christensen *et al.,* 2009; Picouet *et al.*, 2010) or MRI (e.g. Mitchell *et al.*, 2001; Collewet *et al.*, 2005; Monziols *et al*., 2006; Margeta *et al*., 2007; Baulain, 2013) as the reference technology for carcass grading in abattoirs (e.g. Branscheid *et al*., 2011; Daumas *et al.*, 2013) or for performance testing in farm animal breeding programs (e.g. sheep: von Korn *et al*., 2005; Baulain *et al.*, 2011; rabbits: Nagy *et al.*, 2010; Gyovai *et al.*, 2012; Szendrő *et al.*, 2012; pigs: McEvoy *et al*., 2009; Kremer *et al*., 2012, 2013; broiler: Davenel *et al.*, 2000; Milisits *et al.*, 2013; laying hens: Szentirmai *et al.*, 2013; and turkeys: Andrássy-Baka *et al.*, 2003).

Owing to changes in carcass confirmation caused by breeding progress in various farm animal populations, differences among breeds/species themselves or gender-specific carcass composition, there is a steady need for newly derived or adapted formulas for the (S)EUROP classification in carcass grading (Baulain *et al.*, 2003; Branscheid *et al.*, 2011; Monziols *et al.*, 2013) or for performance testing (Tholen *et al.*, 2003; Bernau *et al.*, 2013 and 2015). This is necessary as long as no whole-body or whole-carcass information would be used (Kongsro *et al.*, 2008).

## Non-invasive techniques for body/carcass composition measurements

**Figure 1:**
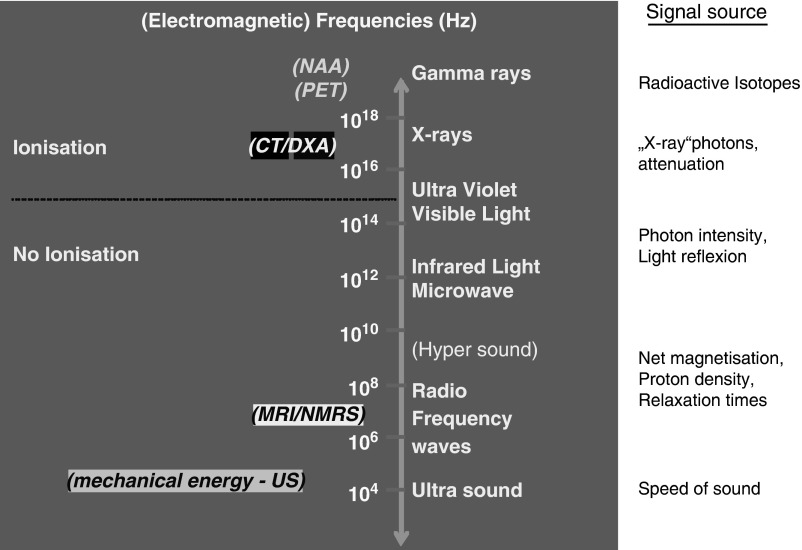
Overview of imaging methods.

A common feature of non-invasive techniques for body or carcass composition measurements is that they work with electromagnetic or mechanical energies, which are able to pass completely or partially through body or carcass tissues such as muscle (lean meat=protein+water), adipose tissue (fat, lipids) and bone. Figure 1 summarizes the different (electromagnetic) energy levels that are being used for a number of non-invasive measurement techniques.

All techniques shown in Figure 1 rely on specific device-driven signals, which interact with tissues in the body or carcass at the atomic or molecular level, resulting in secondary or attenuated signals detected by the instruments and analyzed quantitatively. The signal (energy) produced by the instrument may be in the form of sound waves (US), X-radiation (e.g. CT and DXA) or radio frequency (RF) waves (MRI). The signals detected by these instruments are processed to measure, for example, tissue depths, areas, volumes or distributions of fat, muscle (water, protein) and partly bone or bone mineral (Table 1).

**Table 1:** Traits determined by non-invasive techniques

Non-invasive technique	Trait
Dual-energy X-ray absorptiometry (DXA)	Photon number passage (tissue level)
	X-ray attenuation coefficient from two energy levels
	Bone mineral content
	Bone mineral area
	Bone mineral density
	Soft tissue mass
	Lean tissue mass
	Fat tissue mass
	Whole-body and regional data
Computed tomography (CT)	Photon number passage (tissue level)
	X-ray attenuation in Hounsfield units (HU)
	Tissue areas or volumes depending on anatomical position and HU
	Regional and whole-body data
Magnetic resonance imaging (MRI)	Nuclear magnetic resonance pattern (atomic level)
	Energy level (net magnetization) of nuclei with uneven proton and neutron number
	Longitudinal and transversal relaxation times
	Proton density
	Tissue areas or volumes depending on anatomical position and (arbitrary) signal intensities
	Regional and whole-body data
Ultrasound imaging (US)	Speed of (ultra) sound (tissue level)
	Mechanical energy level *v.* electrical energy level
	Signal amplitude, signal brightness
	Regional distances, areas, volumes

In many cases, these metrology results have to be compared with or validated against a (SI) reference standard directly derived from the carcass. The difference between the arithmetic mean from the non-invasive technique and the arithmetic mean of the reference provides a measure of BIAS or trueness. In addition, the (adjusted) coefficient of determination or regression coefficient (*R*²) combined with an error term or term of uncertainty serves as a statistically defined term of accuracy (i.e. precision). For example, partly depending on the statistical modeling procedure, the root mean squared error of estimation and/or prediction or (cross) validation sometimes standardized as residual standard deviation (root mean squared error divided by the standard deviation of the reference mean) provide information about the uncertainty if compared with a reference technique (Johansen *et al.*, 2007, e.g. Table 2). A standard description is provided by ISO 5725 (https://www.iso.org/obp/ui/#iso:std:iso:5725:-1:ed-1:v1:en). If two techniques are being compared without a ‘gold standard’ or ‘true’ reference, an improved Bland–Altman analysis might be the first choice as the statistical procedure (Liao and Capen, 2011).

**Table 2:** Relationship between carcass composition from dissection and DXA carcass or *in vivo* body composition, depending on species (pig, sheep, cattle) studied (all whole-body DXA data from the same GE Lunar DPX-IQ scanner^1^)

	Pig (*n*=61)			Lamb (*n*=93)			Calf (*n*=34)		
	*R* ^2^ (√m.s.e./s.d.)			*R* ^2^ (√m.s.e./s.d.)			*R* ^2^ (√m.s.e./s.d.)		
Dissection *v.* DXA		Carcass	*In vivo*		Carcass	*In vivo*		Carcass	*In vivo*
Reference	CV			CV			CV		
FAT (%)	0.19	0.80 (0.46)	0.74 (0.46)	0.22	0.73 (0.52)	0.51 (0.69)	0.14	0.28 (0.86)	0.003 (1.02)
FAT (g)	0.28	0.90 (0.32)	0.89 (0.43)	0.31	0.83 (0.42)	0.71 (0.27)	0.23	0.64 (0.78)	0.42 (0.39)
Meat (%)/soft lean (%)	0.05	0.70 (0.57)	0.65 (0.64)	0.04	0.57 (0.66)	0.50 (0.70)	0.04	0.53 (0.69)	0.09 (0.97)
Meat (g)/soft lean (g)	0.14	0.94 (0.39)	0.82 (0.55)	0.10	0.88 (0.35)	0.57 (0.33)	0.23	0.98 (0.13)	0.94 (0.12)

DXA=dual-energy X-ray absorptiometry.

1 This is the only known comparison for the three livestock species using the same DXA device always to compare carcass and *in vivo* data with reference dissection, modified from Scholz *et al.* (2013).

Several publications summarize further invasive or non-invasive methods that are not considered in this review. Simeonova *et al.* (2012), for example, provide a recent review focusing on methods for determining pig body composition, especially on protein deposition during growth. All ‘radiology’ applications reviewed in this paper are based on the inventions and findings of a number of scientists and engineers summarized in the book Classic Papers in Modern Diagnostic Radiology.

### DXA

Both the techniques, CT and DXA, are based on the measurement of the attenuation of X-rays (photons) passing through a body (*in vivo*) or a carcass (*postmortem*). Tissues or elements in the body or carcass are characterized by specific mass attenuation coefficients, depending on the photon energy level being applied for the measurement. DXA and a combination of DXA and CT (dual-energy X-ray computed tomography – DECT; see Johnson, 2009; Magnusson *et al.,* 2011) are based on the application of two different X-ray photon energy levels (high and low), whereas CT works (simplified) only with one (monochromatic) X-ray photon energy level (Kalender, 1988). The ratio (by using the natural logarithm=ln) of the attenuated (*I*) and the initial X-ray photon number (*I*_*O*_) for the low (*L*) and the high (*H*) energy levels provides the so-called *R* value (X-ray attenuation coefficient). This *R* value is – depending on the energy levels used – a unique trait for a certain element or compound tissues, such as bone mineral, soft, lean or fat tissues (Crabtree *et al.*, 2007; Wang *et al.*, 2010). Different generations of DXA (or CT) machines use either pencil or fan-beam technology. The fan-beam technology has been extended to a so-called cone-beam or a flash-beam technique. A whole-body scan with a rather slow but very accurate pencil-beam scanner could take up to 35 min, whereas a whole-body scan with a high-speed cone-beam scanner takes <3 min. Different manufacturers of DXA (or DECT) scanners use different approaches to create a high and a low X-ray energy levels (Ulzheimer and Flohr, 2009). Therefore, DXA needs cross-validation for transferring composition results among devices and software modes (Plank, 2005; Scholz *et al.*, 2007 and 2013; Hull *et al.*, 2009; Lösel *et al*., 2010). In addition, DXA as an indirect tool (Dunshea *et al.*, 2007; Scholz and Mitchell, 2010; Hunter *et al.*, 2011) does not provide a measure of the lean meat percentage. It is still necessary to determine the accuracy of DXA by reference dissection or chemical analysis. The whole-body/carcass composition estimate is available immediately after the scan is completed. Alone, a regional analysis in order to quantify the 2D tissue distribution requires manual manipulation time, depending on the number and anatomical specification of the regions of interest.

DXA studies have been performed on a large variety of farm animal species such as pigs (or pork): Mitchell *et al.* (2000 and 2003); Scholz *et al.* (2002); Suster *et al.* (2004); Marcoux *et al.* (2005); Scholz and Förster (2006); Latorre *et al.* (2008); Kremer *et al.* (2012, 2013); Kogelman *et al.* (2013); chicken/broiler/eggs: Swennen *et al.* (2004); Schreiweis *et al.* (2005); England *et al.* (2012); Salas *et al.* (2012); turkeys: Schöllhorn and Scholz (2007); sheep (or lamb carcasses): Mercier *et al.* (2006); Hopkins *et al.* (2008), Ponnampalam *et al.* (2008); Pearce *et al.* (2009); Hunter *et al.* (2011); Scholz *et al.* (2013); calves/calf carcasses: Bascom (2002); Scholz *et al.* (2003); Hampe *et al.* (2005); fish: Wood (2004), or beef: Ribeiro *et al.* (2011); as well as in the wool and meat industry: Kröger *et al.* (2006) and Ho *et al.* (2013).

The accuracy of DXA measurements comparing pigs, lambs, calves and turkeys has been summarized recently by Scholz *et al.* (2013). To our knowledge, this is the only comparison performed always with the same GE Lunar DPX-IQ (GE Healthcare, Oskar-Schlemmer-Strasse 11, D-80807 München) machine (Table 2). Accuracies for turkeys (*n*=100) measured with the same GE Lunar DPX IQ pencil-beam scanner comparing DXA carcass with chemical analysis data resulted in the following coefficients of determination as are for fat (%): *R* ^2^=0.74 (√m.s.e.=2.11), fat (g): *R* ^2^=0.86 (√m.s.e.=254), protein+water *v.* soft lean (%): *R* ^2^=0.69 (√m.s.e.=2.33) and protein+water *v.* soft lean (g): *R* ^2^=0.99 (√m.s.e.=178) (data from Kreuzer, 2008). The accuracy (low *R* ^2^, high r.s.d.) for lean meat percentage in calves (Table 2) is rather low due to the relatively low variability in the lean meat percentage of the young calves in comparison with the relatively high variability of the lean tissue weight especially *in vivo*. The error (inaccuracy) is even inflated during reference dissection, especially by the individual butcher effect (Nissen *et al.*, 2006). The relatively low absolute amount of fat leads to relatively large errors in percentage values for lean and fat tissues. The main difference among calves originates from different BWs causing variations in lean tissue weight. Bascom (2002) concluded that DXA is not suitable for the prediction of the percentage of carcass fat or carcass CP in Jersey calves (adjusted *R* ^2^<0.1), although it is unclear what was done during that study in terms of the DXA analysis. Dunshea *et al.* (2007) found higher prediction accuracies for chemically determined reference carcass composition in sheep with *R*² of 0.98 for lean weight and of 0.94 for lean percentage. In addition, Hunter *et al.* (2011) stated that DXA-derived estimates of total and individual tissue masses are highly related to, and can be used to predict, chemical composition *in vivo* or of whole carcasses and carcass halves (in sheep). An adjustment of the prediction equations, however, depends in all cases on the manufacturer (General Electrics, Hologic, Norland, Diagnostic Medical Systems), species, age or weight, software mode and animal positioning on the scan table.

### CT

Contrary to DXA and DECT, CT works with only one (monochromatic) X-ray level (Kalender, 2006). The mass attenuation coefficient of the object (tissue) of interest is transformed into the so-called Hounsfield units (HU) or CT values by taking the mass attenuation for water and air into account. The almost-fixed range of HU for a given tissue could be used for (fully) automated image segmentation, distinguishing among the body tissue fat, muscle (water) and bone (Glasbey *et al.*, 1999; Johansen *et al.*, 2007; Bünger *et al.*, 2011; Gjerlaug-Enger *et al.*, 2012; Font-i-Furnols *et al.*, 2013; Jay *et al.*, 2013; Judas and Petzet, 2013; Monziols *et al.*, 2013). There is, however, for *in vivo* studies, some overlap between fat and mammary tissue or fat and lung tissue on one side of the HU scale, and bone or muscle, as well as internal organs such as liver, tumor tissue and blood on the upper side of the HU scale. It has to be considered, additionally, that differences in CT protocols may lead to variations of up to 20% in the HU values, especially for (bone containing) tissues with densities >1.1 g/cm^3^ (Zurl *et al.*, 2014). As described above, tissue segmentation, for example, by threshold setting is based on assumptions of specific mass attenuation coefficients for different body or carcass tissues, which are calculated as HU. It is, however, not always given – not alone depending on the tissue temperature (Szabó and Babinszky, 2008) – that muscle tissue is detected automatically as muscle tissue (or meat≠meat, fat≠fat) when comparing different CT machines using the same individual(s) (Bünger *et al.*, 2011). Besides small variations for non-adipose tissue (HU=+49 to +52), there is variation in CT values or HU of the adipose tissue within growing pigs. The mean adipose tissue HUs for all pigs (*n*=9) in a study by McEvoy *et al.* (2008) were −90, −98 and −101 at mean BWs of 51.4, 93.8 and 124.1 kg, respectively. Owing to the anatomical structure of farm animals (or fish: Nanton *et al.*, 2007; Kolstad *et al.*, 2008), however, CT, like DXA, is very well-suited for the discrimination between bone and soft tissues in sheep, chicken, rabbits, beef including buffalo and goose liver *in vivo* (sheep: Johansen *et al.*, 2007; Kvame and Vangen, 2007; Navajas *et al.*, 2007; Macfarlane *et al.*, 2009; Bünger *et al.*, 2011; Ho *et al*., 2013; chicken: Milisits *et al.*, 2013; Szentirmai *et al.*, 2013; rabbits: Nagy *et al.*, 2010; beef: Hollo *et al.*, 2008; Navajas *et al.*, 2010; buffalo carcass: Holló *et al.*, 2014; goose liver *in vivo*: Locsmandi *et al.*, 2005). Milisits *et al.* (2013) and Szentirmai *et al.* (2013), for example, provided a so-called fat index for determining the body fat content in broiler chicken and laying hens, respectively, by calculating the ratio of the number of fat pixels within the HU range from −20 to −200 to the total number of pixels with HU values for muscle, water and fat between −200 and +200. The muscle index provided additionally by Milisits *et al.* (2013) uses the number of muscle pixels within the HU range from +20 to +200, instead of the fat pixel HU range. The variation found by Chang *et al.* (2011) for various points of visceral and subcutaneous fat in minipigs lies in the range of the above-defined HU threshold values for ‘chicken’ fat (−20 and −200), with −108.80±5.77 as the lowest mean value (±s.d.) for subcutaneous fat and with −119.41±6.90 as the highest HU value for visceral fat. Johansen *et al.* (2007) provided the following HU thresholds for tissue segmentation in lambs: bone *v.* soft tissue ‘kC’=296; soft tissue *v.* background noise (air) ‘kA’=−156 and fat *v.* muscle ‘kB’=10. The sum of pixels within these thresholds served as estimates of fat and muscle tissue, although according to the thresholds mentioned water was included into the fat tissue.

The latest machines are now the so-called multi-slice spiral (or helical) CTs based on a rotating X-ray source and an array of X-ray photon sensors on the opposite side of the CT gantry (Ulzheimer and Flohr, 2009). Especially for CT, the development of technology occurs at a breathtaking speed. It took only about 10 years from single-slice to multi-slice machines to be developed (Kopp *et al.*, 2000), with now more than 100 slices for one rotation. The body region covered increased from about 1 cm to more than 10 cm/s, whereas the minimal slice thickness decreased from 5 mm to <0.5 mm at the same time (Kalender, 2006). In addition, the gantry size now reaches up to 90 cm providing space for bigger (heavier) farm animals.

After semi-automatic image analysis using OsiriX (Rosset *et al.*, 2004) or ATAR (Animal Tomogram Analysis Routines) software (Haynes *et al.*, 2010; Bünger *et al.*, 2011; Jay *et al.*, 2013), fat, muscle and bone areas can be calculated within the slices of interest. The traits (phenotypes) calculated serve as the basis for the prediction of carcass and tissue weights or volumes and proportions of muscle, fat and bone in combination with additional linear measurements for 2D gigot muscularity, loin eye muscle area and 2D loin eye muscularity and finally as basis for breeding value estimation (Bünger *et al.*, 2011). Present developments aim at whole-body spiral scanning in order to measure the above traits instead of having to predict them, leading additionally to 3D gigot muscularity and 3D loin eye muscularity. The 3D information can even help to include the retailer into the development of new products by applying ‘PorkCAD’, a new ‘design butcher’ (Virtual Slaughterhouse) system based on a virtual pig created from CT scanning as suggested by Laursen *et al.* (2013).

Somewhat different approaches and assumptions among the European colleagues from Denmark, France, Hungaria, Ireland, Norway, Sweden, Spain and United Kingdom led to different solutions for carcass grading, and especially formulas for the prediction of the lean meat percentage, which is or should be the basis for the payment of producers (Szabó and Babinszky, 2009). There is, for example, a discussion going on whether meat yield should be determined on the basis of scale weight or on the basis of CT volume (Olsen and Christensen, 2013 *v.* Daumas *et al.,* 2013). Scale weight would require assumptions or knowledge about the ‘true’ CT density of lean meat (Daumas *et al.*, 2013). Differences in the calculation of CT densities for lean meat result in different lean meat weights for similar lean meat volumes, making the harmonization among different countries or among various CT scanners more complicated (Daumas *et al.*, 2013; Olsen and Christensen, 2013).

Correspondingly, CT studies *post mortem* are also aiming to determine the salt content in the dry-cured ham, because the changing NaCl and H_2_O proportions lead to modified X-ray attenuations (Fulladosa *et al.*, 2010); whereas Frisullo *et al.* (2010) used micro-CT for the rapid estimation of intramuscular fat (IMF) in beef and for the description of the fat microstructure. Anton *et al.* (2013) compared chemical analysis or dissection with CT in order to determine the IMF and carcass fat content of beef in a study focusing on the thyroglobulin (TG) polymorphism. They calculated correlations between IMF (% from Soxhlet analysis) and CT fat (%) in *musculus longissimus dorsi*, and between dissected fat (%) of the right carcass-half and CT fat (%) between the 11^th^ and 13^th^ rib joint of 0.71 and 0.96 (*P*<0.001), respectively. In this context, Jose *et al.* (2009) stated that CT scanning does not negatively affect the quality of (beef or lamb) meat, especially in terms of color. Kongsro and Gjerlaug-Enger (2013) – in pigs – and Clelland *et al.* (2013) – in sheep – started using CT for the measurement of meat quality (IMF content) *in vivo*. The regression coefficients between CT IMF *in vivo* (+further variables) and IMF in the carcass loin eye reached values of adjusted *R* ^2^⩽0.71 (r.m.s.e. ⩾0.36) for Texel lambs, whereas a significantly lower relationship between IMF and CT intensity values was found (*R* ^2^=0.18; RMSEP=0.48) according to Kongsro and Gjerlaug-Enger (2013) in pigs. In contrast to the US study by Jiao *et al.* (2014), the relatively low level of IMF and small variation in the Duroc boars studied in comparison with ordinary slaughtered pigs may have led to low prediction accuracies based on CT signal intensities. Font-i-Furnols *et al.* (2013) describe a further method to determine IMF in pork loins using CT. The best prediction of IMF resulted from ordinary linear regression analysis when data from two tomograms were used (*R* ^2^=0.83 and RMSEPCV=0.46%). However, genomic selection for IMF improvement based on NIR derived IMF might be a more promising approach according to Gjerlaug-Enger *et al.* (2014).

Deeper insights into the physiological role of IMF in comparison with intermuscular fat (adipose tissue) are provided by Hausman *et al.* (2014).

### MRI

The principle of MRI relies on the net magnetization of spinning nuclei with uneven proton and neutron numbers and RF-induced 3D-coded voltage readings with tissue-specific relaxation times depending on spin-lattice (T1) and spin–spin (T2) interactions combined with the proton density (Laurent *et al*., 2000; Baulain and Henning, 2001; Mitchell *et al*., 2001). In addition, T1 and T2 depend on the magnetic field strength (Kato *et al*., 2005). Furthermore, the effect of dehydration plays a crucial role not alone in (dry) cured ham and can be used for MRI applications by taking advantage of changing T1 and T2 relaxation times, which depend on the salt content in the ham (Fantazzini *et al*., 2009).

**Figure 2:**
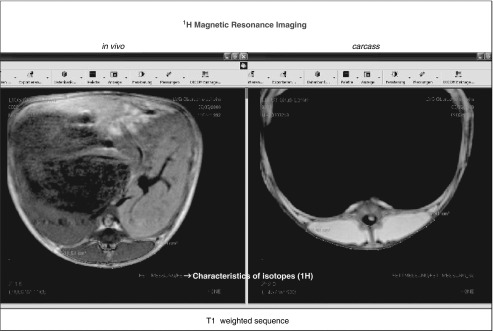
Differences in NMR proton characteristics depending on body temperature (left: lamb *in vivo* ~37°C, right: lamb carcass chilled <8°C, free software DicomWorks, ^©^Philippe PUECH).

A combination of magnetic field produced either by a ferromagnetic, electromagnetic or superconducting system with a field strength between 0.1 and 7 T and so-called gradient coils with a corresponding RF frequency (Larmor frequency) sequence creates a number of cross-sectional images with a 3D voxel definition for the *x*-, *y*- and *z*-axis direction. A Fourier transformation helps in recalculating the signal information from the spectral domain into pixel (or voxel)-wise signal intensity values in a ‘gray scale domain’ visible on the ‘computer’ screen. For a T1-weighted sequence with a TR (time between two consecutive RF pulse signals or between successive excitations) of 300 ms and a TE (time between echoes=between middle of exciting RF pulse signal and middle of spin echo production) of 17 ms, the fat tissue containing pixels have rather high signal intensities, whereas the non-fat pixels show lower signal intensities. This pattern, however, changes on chilled objects (Monziols *et al*., 2005 and 2006). As shown in Figure 2, a T1-weighted sequence would show dark pixels (low signal intensity) for fat tissue and brighter pixels for lean meat tissue (relatively higher signal intensity).

The above-mentioned Larmor (resonance) frequency differs depending on the isotope of interest and the magnetic field strength. Because the isotope ^1^H has the largest relative sensitivity and highest natural frequency compared with ^2^H and ^3^H, proton or ^1^H nuclear MRI is the most often used method, and is even applied for the study of pork pie (Gaunt *et al*., 2013).

Usually, an MRI or also a CT (DECT) scan starts with a so-called scout or localizer image sequence in order to be able to define the ‘slice’ positions and directions as targeted. After successful image acquisition and data storage, a quantitative image analysis is required in order to measure – either in the most simple way – the regions of interest (distances or areas) or calculate – after a more challenging segmentation procedure – the volumes of interest relevant for body or carcass composition measurements. Based on T1 mapping, Kullberg *et al.* (2006 and 2007), for example, described a fully automated protocol for MR image analysis, focusing on the segmentation of visceral and subcutaneous fat in humans, whereas Addeman *et al.* (2015) suggested a so-called fat fraction mapping for the automatic determination of subcutaneous adipose and intra-abdominal adipose tissue within the total adipose tissue.

**Figure 3:**
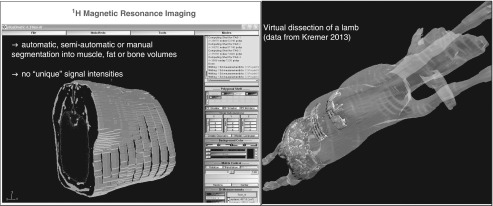
Examples for image analysis and 3D re-calculation (left software used: sliceOmatic, Tomovision Inc.; right software used: 3D DOCTOR, Able Inc., data from Kremer, 2013).

Various free or commercial software packages are available in order to automate image segmentation into muscle/lean meat, fat, bone and, if necessary, gastrointestinal tract/abdominal content (Figure 3). This procedure can be standardized more easily for CT images than for MRI images, because of the ‘unique’ application of HU for tissues like bone, muscle (water) and fat. Signals within MR images depend on the tissue-specific relaxation times T1 and T2, including proton density, and on various technical conditions and sequence settings such as the magnetic field strength, the RF pulse sequence(s), slice thickness, distance between slices, number of acquisitions and the specification of (body) coil used.

A relatively new non-invasive (but non-imaging) method ‘QMR’ – quantitative magnetic resonance – is still in the evaluation phase for farm animals (Mitchell *et al.*, 2012).

### US scanning/imaging

Information retrieval by measuring the velocity of sound is the only method among the methods described in this study that depends on mechanical energy fluctuations. The general principle is based on the partial reflection of (longitudinal) US waves from the interface between different media and/or body tissues (>20 kHz, Scholz and Baulain, 2009; Halliwell, 2010; Scholz and Mitchell, 2010; Pathak *et al.*, 2011).

Different tissues have different (sound) attenuation coefficients depending on the frequency for the creation of the US (waves), whereas the speed of longitudinal sound waves increases with the density of the material the sound wave is travelling through (Halliwell, 2010; Culjat *et al.*, 2012). Because the density of (body) tissues is also temperature-dependent, it makes a difference if a (chilled) carcass or a living animal is ‘ultrasonographed’. The speed of US is 1403 m/s in water of a temperature of 0°C and 1472 m/s in water of a temperature of 17°C (Vogt *et al.*, 2008). Van de Sompel *et al.* (2012) obtained a calculated speed of US of 1524 m/s in water with a temperature of 37°C for a salinity of 0% at 0 meter below water.

Because of the accelerated technical improvement of real-time linear-phased array ultrasonic transducers and scanners, this technique has become the most common technology for (farm) animal body and carcass composition assessment (Starck *et al*., 2001; Mitchell and Scholz, 2009, Scholz and Baulain, 2009).

**Figure 4:**
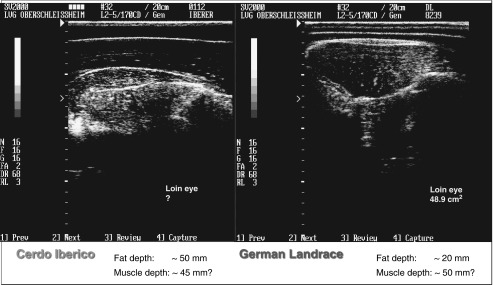
Comparison of ‘obese’ and ‘standard’ pigs (using a variable 2.5 to 5 MHz ‘backfat’ 17-cm transducer).

Two-dimensional US images from so-called B-mode (brightness) devices provide information about adipose tissue depots and cross-sectional areas of muscles, whereas A-mode devices (amplitude) can be used for simple distance measurements of fat or muscle (meat) layers. Real-time B-mode information (2D or 3D images) result from rapid electronic switching or phased array transducers (a number of piezoelectric elements) of different shapes (Starck *et al*., 2001). At present, most of the US devices for performance testing use (linear) phased array transducers to convert electronic energy to high-frequency ultrasonic (mechanical) energy that travels through the animal body in short pulses. As soon as ultrasonic waves meet at an interface between two tissues that differ in acoustical properties, a part of the (longitudinal) ultrasonic waves are reflected back to the receiver probe (the phased array transducer). Variations in fat, muscle or bone tissue depths or in the distribution of, for example, intermuscular and especially IMF result in time differences in reflected ultrasonic wave signals affected additionally by absorption and refraction (scatter) of the mechanical energy (Starck *et al.*, 2001). These effects combined with variations caused by the expertise of the testing person, age/weight of the animal and the behavior of the animal tested lead in some cases to a challenging interpretation of the US imaging or scanning results, as can be seen from Figure 4. They make the measurement of areas or even volumes (weights) less accurate in comparison with MRI or CT. Depending on the transducer and on the scan settings in terms of frequency, it might be the case that the measurement of, for example, the loin eye area becomes almost impossible and requires a lot of educated ‘anatomical’ guessing in order to provide reasonable data (Figure 4). Related to the above measurement site on the (beef) animal, Harangi (2013) stated for Charolais bulls that the relationship between ultrasound rib eye area (UREA) and ‘planimeter’ carcass rib eye area (CREA) was higher when measured between the 12^th^ and 13^th^ rib instead of between the 11^th^ and 12^th^ rib with *R* ^2^ of 0.91 and 0.84 (CV 2.16% *v.* 5.3%), respectively. Török *et al.* (2009) found slightly modified relationships between UREA and CREA for four different beef breeds (Limousin *R* ^2^=0.92, Charolais *R* ^2^=0.64, Angus and Simmental *R* ^2^=0.55).

It must always be considered that the attenuation of ultrasonic energy increases with a rising frequency, whereas the tissue penetration depth of the ultrasonic energy waves decreases with increasing attenuation. Therefore, probes with a frequency between 2 and 5 MHz will be used for measurements, including muscle depth or muscle area (volume), whereas probes with frequency >5 MHz (up to 7.5 MHz) will be used for ‘subcutaneous’ scanning where deeper muscle regions are not of interest (Schröder and Staufenbiel, 2006; Pillen and van Alfen, 2011).

As muscle tissue has a higher US attenuation than fat tissue, US technology is widely used in farm animal performance testing (Stouffer, 2004; Pathak *et al.*, 2011; Ayuso *et al.*, 2013), obesity diagnostics (Barbero *et al.*, 2013), body condition scoring (Schröder and Staufenbiel, 2006) or for carcass grading (Branscheid *et al.*, 2011).

## Application in existing breeding programs

The above-mentioned practical applications of US measurements of live animals and carcasses are being extended to genetic selection programs (Müller and Polten, 2004; Kleczek *et al*., 2009; Emenheiser *et al.*, 2010; Case *et al.*, 2012; Maximini *et al.*, 2012; Harangi, 2013), by including *in vivo* IMF (uIMF) measurements in Duroc pigs (Maignel *et al.*, 2010), and, for example, in Angus cattle (Ravagnolo *et al.*, 2010), Nelore cattle (Bonin *et al.*, 2010) or Angus–Brahman crossbred cattle (Elzo *et al.*, 2010).

As ‘…heritability is a simple dimension less measure of the importance of genetic factors in explaining the differences between individuals, and it allows an immediate comparison of the same trait across populations and of different traits within a population’ (Visscher *et al.*, 2008), we use that (additive) genetic variance indicator in the following comparisons of different traits derived from non-invasive techniques (Tables 3 and 4). Heritability estimates for uIMF were rather low with *h* ^2^=0.12 for Angus in Urugay (Ravagnolo *et al.*, 2010) in contrast with very high (most likely overestimated) *h* ^2^ of 0.78 for a very variable Angus–Brahman population in Florida (Elzo *et al.*, 2010). Suther (2009) summarizes a number of earlier studies and shows a similar range of heritability estimates for marbling (IMF) in beef cattle (Table 3).

**Table 3:** Examples of heritability estimates (*h* ^2^, s.e.) for intramuscular fat determined by US *in vivo*

Non-invasive Trait	Technique	Species	Breed	*h*² (s.e.)	Reference
Intramuscular fat (%)	US	Cattle	Angus	0.12 (0.03)	Ravagnolo *et al.* (2010)
			Angus–Brahman	0.78 (0.09)	Elzo *et al.* (2010)
			Diverse	0.12−0.88	Suther (2009)^1^
		Pig	Duroc	0.54 (0.11)	Jiao *et al.* (2014)

US=ultrasound imaging.

1 References before 2010 in Supplementary Material S1.

**Table 4:** Examples of heritability estimates (*h*², s.e.) for body composition traits determined by DXA, CT or US^1^

Non-invasive trait	Technique	Species	Breed	*h*² (s.e.)	Reference
Lean meat (%)	CT	Pig	Duroc	0.57 (0.05)	Gjerlaug-Enger *et al.*, 2012
			Landrace	0.50 (0.05)	
Fat (%)	DXA	Pig	F2 with Göttinger Minipig	0.57 (0.14)	Kogelman *et al.*, 2013
Lean Meat (g)	CT	Sheep	Charolais, Suffolk, Texel	0.47, 0.45, 0.46 (0.09)	Jones *et al.* (2004)^2^
			Norwegian White	0.57 (0.16)	Kvame and Vangen (2007)
			Scottish Blackface	0.48 (0.17)	Karamichou *et al.* (2006)
	DXA	Pig	F2 with Göttinger Minipig	0.71 (0.14)	Kogelman *et al.* (2013)
Fat (g)	CT	Sheep	Charolais Suffolk, Texel	0.38, 0.41, 0.40 (0.09)	Jones *et al.* (2004)
			Norwegian White	0.29 (0.13)	Kvame and Vangen (2007)
			Scottish Blackface	0.6 (0.28)	Karamichou *et al.* (2006)
	DXA	Pig	F2 with Göttinger Minipig	0.43 (0.13)	Kogelman *et al.* (2013)
Bone mineral (g)	DXA	Pig	F2 with Göttinger Minipig	0.76 (0.15)	Kogelman *et al.* (2013)
Bone mass (g)	CT	Sheep	Norwegian White	0.51 (0.15)	Kvame and Vangen (2007)
			Scottish Blackface	0.14 (0.11)	Karamichou *et al.* (2006)
Bone mineral density (g/cm²)	DXA	Pig	F2 with Göttinger Minipig	0.92 (0.16)	Kogelman *et al.* (2013)
Loin eye area (cm²)	CT	Sheep	Scottish Blackface	0.33 (0.12)	Karamichou *et al.* (2006)
			Five diverse Austrian	0.24 (0.03)	Maximini *et al.* (2012)
	US	Cattle	Nellore	0.31 to 0.34 (0.03)	Caetano *et al.* (2013)
			Hanwoo	0.09 to 0.24 (0.09 to 0.16)	Lee *et al.* (2014)
		Pig	Duroc, Landrace, Yorkshire	0.21 to 0.22 (<0.01)	Choi *et al.* (2013)
Fat area (cm²)	CT	Sheep	Scottish Blackface	0.5 to 0.76 (0.08 to 0.22)	Karamichou *et al.* (2006)
			five diverse Austrian	0.36 to 0.4 (0.03)	Maximini *et al.*( 2012)
Muscle depth (mm, cm)	US	Sheep	Charolais Suffolk, Texel	0.30, 0.32, 0.29 (0.01 to 0.02)	Jones *et al.*, 2004
			Norwegian White	0.28 (0.05)	Kvame & Vangen, 2007
			five diverse Austrian	0.28 (0.05)	Maximini *et al*. (2012)
			Broiler commercial line	0.28 to 0.51 (0.02)	de Genova Gaya (2013)
		Pig	Duroc	0.39 (0.09)	Jiao *et al*. (2014)
Fat depth (mm, cm)	US	Sheep	Charolais, Suffolk, Texel	0.34, 0.35, 0.38 (0.01 to 0.02)	Jones *et al*. (2004)
			Norwegian White	0.44 (0.06)	Kvame and Vangen (2007)
			five diverse Austrian	0.29 (0.05)	Maximini *et al*. (2012)
		Cattle	Angus	0.26 to 0.46 (0.03 to 0.08)	MacNeil and Northcutt (2008)
			Nellore	0.23 (0.02)	Caetano *et al*. (2013)
			Hanwoo	0.05 to 0.47 (0.06 to 0.15)	Lee *et al*. (2014)
		Pig	Duroc	0.58 (0.09)	Jiao *et al*. (2014)
			Duroc, Landrace, Yorkshire	0.32, 0.41, 0.38 (<0.01)	Choi *et al*. (2013)

CT=computed tomography; DXA=dual-energy X-ray absorptiometry; US=ultrasound.

1 Many more studies with ‘US’ heritability estimates exist.

2 References before 2010 in Supplementary Material S1.

Expectedly, real-time US data of muscle depth in sheep or breast muscle thickness in broilers showed medium-to-high direct heritability estimates between 0.2 and 0.51 (Jones *et al.*, 2004, Wolf and Jones, 2007; Grosso *et al.*, 2010, Maximini *et al.*, 2012; Table 4), whereas heritability estimates – depending on age – varied in a similar range between 0.31 and 0.42 for loin muscle (or rib eye) area (between 12^th^ and 13^th^ ribs) in *Bos indicus* (Bonin *et al.*, 2010; Pinheiro *et al.*, 2011), Angus–Brahman (Elzo *et al.*, 2010) and multi-breed beef cattle (Jeyaruban and Johnston, 2014). Heritability estimates for back fat thickness (between 12^th^ and 13^th^ ribs) showed slightly lower values ranging from 0.06 to 0.32 (Bonin *et al.*, 2010; Elzo *et al.*, 2010; Pinheiro *et al.*, 2011) as well as for rump fat thickness with values from 0.26 to 0.29 (alone Pinheiro *et al.*, 2011). The advantage of US scanning can be concluded from the above references. US is the only method among the reviewed ones that can be applied in (beef) cattle (Drennan *et al.*, 2009) without size restrictions as there exist for CT, DXA and MRI.

Besides US, CT alone is being used in practical farm animal breeding programs, especially for the selection of body composition traits in pigs and sheep. In this context, CT is, meanwhile, declared as ‘part of the routine genetic selection programs in modern times’ (Ley, 2013). This, however, is true for only a very few breeding organizations or CT service (research) units in the world so far – such as, for example, for sheep selection in Australia, New Zealand and United Kingdom (e.g. Lambe *et al.*, 2008; Arthur *et al.*, 2011, Bünger *et al.*, 2011) or pig (and sheep) selection in Norway (e.g. Kvame *et al.*, 2006; Kongsro *et al.*, 2008; Gjerlaug-Enger *et al.*, 2012). Gjerlaug-Enger *et al.* 2012 estimated heritabilities for CT lean meat percentage (LMP) between 0.5 and 0.57 (Table 4).

Therefore, the available high additive genetic variance for lean meat percentage in both Norwegian pig breeds based on *in vivo* whole-body CT measurement makes that technique very efficient for selection decisions without having to sacrifice potential breeding animals. At present, the capacity of modern CT machines allows the acquisition of more than 1100 slices per farm animal (e.g. male or female breeding pigs) in an actual whole-body scanning time of less than a minute *in vivo* (Gjerlaug-Enger *et al.*, 2012). Handling, scanning and image analysis for one potential breeding boar or gilt under performance testing takes, in the meantime, only about 15 min. Image analysis is fully automated using MatLab^®^ (The MathWorks Inc., Natick, MA, USA) procedures, especially adapted to CT volume information. A total of 24 boars tested per day is a routine application at Topigs-Norsvin facilities. Information from 1100 slices per potential breeding boar are processed for the body composition phenotypes like lean meat (kg, %), fat (kg, %), bone (kg, %), primal cuts (kg), live and ‘carcass’ weight (kg), as well as carcass yield (%) (Gjerlaug-Enger *et al*., 2012).

Slightly modified approaches serve for sheep selection programs at the SRUC (Scotland, UK) in Edinburgh. Routine application at the SRUC covers three important body regions such as thorax (transversal slice at thoracic vertebra 8), loin (lumbar vertebra 5) and gigot (ischium – back of the pelvis) of breeding sheep (Bünger *et al.*, 2011).

Maximini *et al.* (2012) compared genetic (across breeds) parameters for phenotypes derived from either CT or US in five Austrian sheep breeds. They found moderate *h*² estimates for US scan traits for eye muscle depth (0.28) and for fat depth (0.29), whereas CT traits showed higher (across breed) *h* ^2^ estimates for fat (0.36 and 0.40), but not for the eye muscle area (0.24) (Table 4). Among other unknown reasons, these slightly problematic across-breed heritability estimates (Visscher *et al.*, 2008) led Austrian sheep breeders to abandon CT in favor for US (Fürst-Waltl and Grill, 2013).

In contrast with Maximini *et al.* (2012), Karamichou *et al.* (2006) found, for all CT tissue areas, moderate-to-high heritability estimates between 0.23 and 0.76. The heritability estimates for CT fat areas started at 0.5, whereas the estimates for loin eye muscle area showed an average value of 0.33 for the univariate model (Table 4). Meanwhile, sheep breeders from New Zealand advocated the combination of US and CT in a stage breeding design because ‘selection of meat sheep on CT measurements will increase genetic progress, compared with selection on US measurements alone’ (Bünger *et al.*, 2011). In this context, Moore *et al.* (2011) demonstrated that, for sheep, combining CT with US scanning would increase the estimated breeding value (EBV) accuracy by 6% to 20% in comparison with US scanning alone, thus supporting the great benefit of CT. These calculations are derived from US prediction accuracies (*R* ^2^) in the order of 0.65 and 0.50 for fat (kg) and muscle (kg), respectively, with heritabilities for US-measured muscle and fat depth of 0.24 to 0.32 and 0.19 to 0.38, respectively (Jones *et al.*, 2004; Bünger *et al.*, 2011; Mortimer *et al.*, 2014), whereas accuracies of CT-based predictions in meat sheep for fat and muscle weight are significantly higher with *R* ^2^=0.99 (r.s.d.=434 g) and 0.97 (r.s.d.=611 g), respectively, combined with expected corresponding heritabilities between 0.4 and 0.5 (Young *et al.*, 2001) or between 0.35 and 0.45 (Jones *et al.*, 2004).

Neither DXA nor MRI are being actively used in commercial breeding programs so far, although Kogelman *et al.* (2013) estimated ‘heritability’ estimates in a F2 pig population originating from crosses of Duroc or Yorkshire and Göttinger Minipig. The heritability estimate for DXA lean mass, of 0.71, is higher than that for DXA fat mass, of 0.43. This observation corresponds with the order of the sheep CT heritability estimates for lean and fat mass, with the exception of *h*² estimates for the Scottish Blackface (Table 4).

## Harmonization and comparison of techniques

Reference (volume) phantoms could help making different CT machines comparable (Christensen and Angel, 2013). The same is true for DXA, because different machines within or among different manufacturers use various settings in order to measure the X-ray attenuation coefficient (*R* value) based on the specific X-ray attenuation of body tissues for high- and low-energy levels (Wood, 2004; Plank, 2005; Lösel *et al.*, 2010). It is even more difficult with MRI, because there are no standardized signal intensities describing one or the other tissue (Baulain and Henning, 2001; Mitchell *et al*., 2001; Kremer *et al.*, 2012 and 2013; Collewet *et al.*, 2013; Addeman *et al.*, 2015; Pérez-Palacios *et al.*, 2014). In addition, not only the velocity of sound depends on the surrounding temperature of body/carcass tissues but also the attenuation of X-rays (Szabó and Babinszky, 2008), and the electromagnetic patterns of protons lead to different results for *in vivo* and *postmortem* (carcass) measurements within the same animal.

The objective of all imaging techniques is to achieve an optimum signal-to-noise ratio combined with small voxel sizes for discrete image segmentation into the body tissues (structures) of interest (Hanna and Cuschieri, 2001). Chemical shift or partial volume effects must be considered when interpreting the accuracy of MRI-derived body/carcass composition estimates (Monziols *et al.*, 2005). Monziols *et al.* (2006) found an increase in estimation accuracy (higher *R* ^2^, lower residual standard deviation) for muscle or fat weight and percentage with an increase in body regions (slices) analyzed. If the most relevant body regions are accounted for, or even the whole body, according to the Cavaleri method, MRI, like CT (and DXA), is a very useful tool for growth- or obesity-related studies, because there is no need for serial slaughter anymore (Cavaleri method: Gong *et al.*, 2000; Glasbey and Robinson, 2002; Baulain *et al.*, 2003; Mandarim-De-Lacerda, 2003; Vogt *et al.*, 2007; Szabó and Babinsky, 2009; Arthur *et al.*, 2011).

Only a very few studies exist where DXA measurements are being compared with MRI (Vogt *et al.*, 2007; Brandberg, 2009; Bernau *et al*., 2015). Vogt *et al.* (2007) found a relationship of *R* ^2^=0.95 between whole-body fat measurements performed by DXA (pencil-beam scanner) and by MRI (1.5 T) in human probands, whereas DXA (GE Lunar DPX-L) underestimated the total fat weights compared with both CT −5.23 kg (1.71 kg) and MRI −4.67 kg (2.38 kg) in another study on human probands, summarized in the thesis by Brandberg (2009). Bernau *et al.* (2015) showed for 20 intact boars that both, MRI and DXA, can be used with high accuracy (*R* ^2^=0.88 or 0.91, r.m.s.e.=0.9% or 0.82%, respectively) to predict lean meat percentage from dissection. The combination of both techniques resulted in an *R* ^2^ of 0.95 (with r.m.s.e.=0.61%). Mitchell and Scholz (2009) could additionally show that the relationship between DXA measurements and the corresponding reference carcass traits was higher than the relationship between US measurements and the same corresponding reference carcass traits with a correlation of *r*=−0.85 (−0.87) for DXA lean % *v.* carcass fat % in comparison with a corresponding relationship for US fat-free mass % *v.* carcass fat %, with *r*=−0.69 (−0.74) for pigs with a BW of 110 or 100 kg, respectively. These findings were supported by Suster *et al.* (2004), who reported that DXA measurement values are more closely related with chemically determined carcass values than are carcass P2 back fat measurements performed using a ruler. DXA (or whole-body/carcass MRI/CT) covers the total amount or percentage of body fat or lean meat, whereas US- or ruler-based back fat measurements can only account for subcutaneous fat layers on a limited number of body regions, and therefore cannot account for all the differences in the distribution of fat layers when comparing different farm animal breeds or genotypes, for example.

Table 5 summarizes studies with the ‘closest’ published relationships between lean meat percentage (LMP) from dissection and the four techniques (DXA, CT, MRI and US) reviewed in this study. All data originate from pigs (carcasses/*in vivo*) for two reasons. First, pigs still have a relatively high variability in body or carcass composition, especially in the subcutaneous fat layer, and, second, reasonable data for the four techniques in the review exist mainly for pigs, followed by sheep and to some extent by poultry or fish.

**Table 5:** Comparison of non-invasive techniques (reference: lean meat % from dissection)

		Accuracy (alone for pigs)					
		*R* ^2^	r.m.s.e.	*R* ^2^	r.m.s.e.		
Method	Reference tissue	Carcass		*In vivo*		Scan time whole body	X-radition exposure (mrem)
CT	Lean meat (%)	<0.99	>0.54	<0.94	>1.00	5 to 30 s	9 to 15
MRI	Lean meat (%)	<0.98	>0.62	<0.87	>1.20	15 to 30 min	None
DXA	Lean meat (%)	<0.91	>0.82	<0.72	>1.75	7 to 13 min	0.03 to 0.06
US	Lean meat (%)	<0.77	>0.70	<0.53	>1.95	–	None

CT=computed tomography; MRI=magnetic resonance imaging; DXA=dual-energy X-ray absorptiometry; US=ultrasound. Carcass: CT data from Judas *et al.* (2005), Romvari *et al.* (2006), Vester-Christensen *et al.* (2009), Monziols *et al.* (2013); MRI data from Baulain and Henning (2001), Mitchell *et al.* (2001), Baulain *et al.* (2003), Collewet *et al.* (2005); Monziols *et al.* (2006); DXA data from Bernau *et al.* (2015); Dunshea *et al.* (2007) (ewes and wheters: % chemical lean: *R* ^2^=0.94); and US data from Branscheid *et al.* (2011). *In vivo*: CT data from Romvari *et al.* (2005) (no error terms provided); MRI data from Baulain and Henning (2001) (*R* ^2^=0.91, r.m.s.e.=1.90% in lambs), Mitchell *et al.* (2001), Scholz (2002); DXA data from Scholz *and Förster* (2006), Mitchell *et al.* (2002) (pigs: *R*²=0.84 for chemical lean %); and US data from Youssao *et al.* (2002), Doeschl-Wilson *et al.* (2005). References before 2010 in Supplementary Material S1.

## Conclusion for imaging/non-invasive methods

If sufficient automatic procedures are available, the ‘Cavalieri’ method, or even better a whole-body scan, is the preferred CT or MRI imaging procedure, because whole-body information does not require breed, species or age-/weight-specific prediction equations.

For performance testing, a combination of the various methods listed below might be optimal based on cost and accuracy:

(1) If radiation and the high investment price are not an issue, then use a ‘New Generation’ spiral, multi-slice CT for the measurement of body/carcass composition.
(2) If 3D information (e.g. carcass cuts, muscle or fat volumes) is not required, use DXA.
(3) If radiation is an issue, use MRI.
  Anesthesia is required in most cases (1–3)!
(4) If a ‘quick’ and ‘easy’ answer is the objective, use A-mode US and for little more B-mode.
  In all cases (1–4), a scale is very useful!

According to Kallweit (1992), one could still conclude that ‘There are advantages and disadvantages of individual systems in their present state...’ as summarized in Table 6. ‘...The rapid progress in technical development may lead to further improvements in the future.’ Actually, nothing has changed in the past 20 years .

**Table 6:** Advantages and disadvantages of non-invasive techniques for the determination of body or carcass composition

	Advantages	Disadvantages
Dual-energy X-ray absorptiometry (DXA)	Easy handling Low radiation Medium price Quick data analysis Regional data analysis	Alone 2D information (so far) No direct data for lean meat (*in vivo*)
Computed tomography (CT)	Very high anatomical resolution High speed Whole-body 3D data Automatic data analysis	X-radiation exposure Expensive
Magnetic resonance imaging (MRI)	Excellent soft tissue differentiation Whole-body 3D data Functional imaging No radiation	Expensive (if high field strength magnet) Rather slow (whole body) Availability (farm animal sector)
Ultrasound imaging (US)	Portable, extensive database for some species Reasonably priced No radiation Real time, online No size limit, no sedation/anesthesia	Less accurate anatomical resolution Image analysis not easily automated No whole-body information

### Present and potential future applications – non-invasive measurements of new exactly measured ‘phenotypes’ to be associated with new ‘genotypes’ and/or fairer payment types

The future of non-invasive techniques or imaging will certainly consider ‘new’ phenotypes, which are of interest for animal breeders, and need attention for next generations of farm animals, such as lean meat and fat deposition efficiency (Martinsen *et al.*, 2014). Most often these will be traits, which could not be recorded routinely without the application of non-invasive techniques like, for example, traits related to leg health. New selectable leg health traits could be bone mineral content or bone mineral density derived by DXA and/or CT (Charuta *et al.*, 2012; Laenoi *et al.*, 2012; Rangkasenee *et al.*, 2013, Rothammer *et al.*, 2014) or osteochondrosis scores as suggested by Aasmundstad *et al.* (2013), with a promising heritability estimate of 0.31 (±0.09). Several groups are already trying to implement meat and partially fat quality (water proportion in fat) measurements *in vivo*. Beef cattle breeders have been using US imaging in order to measure muscle marbling for several years now, whereas CT scanning is being studied in order to measure the IMF content in sheep and pigs during performance testing *in vivo*, without sacrificing the potential high EBV sire or dam breeding animals. More futuristic, but not less relevant, traits could be the volume of (internal) organs as an indicator of the metabolic capacity of breeding animals or a number of morphological traits under indirect selection pressure by present or future breeding objectives (Kongsro personal communication 2012 to 2014, Carabús *et al.*, 2013). Bünger (personal communication 2013 and 2014) suggests to including more 3D information about muscularity of the gigot, as well as the *longissimus dorsi* muscles or other body parts, for UK sheep breeding programs. Other traits could be, for example, the number of vertebrae counted using CT (Donaldson *et al*., 2013), the gut or rumen size as an indicator of greenhouse gas output (Goopy *et al.*, 2014) and, for example, pelvic dimensions as indictors for ease of lambing.

In addition, the combination of exact phenotypic data derived from non-invasive techniques in combination with (whole) genome data will provide more knowledge and deeper insight into the control of growth and body/carcass composition of farm animals (Cavanagh *et al.*, 2010; Rothammer *et al.*, 2014). In particular, phenotypic traits, which are difficult and expensive to measure as the ones derived from the non-invasive techniques discussed in this review, will provide extra value for genomic selection (Hayes *et al.*, 2013).

In general, it seems that non-invasive (imaging) methods have become common practice in the growing scientific community and partly in breeding organizations and abattoirs, like the development of an on-line CT for carcass classification in Denmark.

## Footnotes

1 References before 2010 in Supplementary Material S1.

## Supplementary material

For supplementary material accompanying this paper visit http://dx.doi.org/10.1017/S1751731115000336. click here to view supplementary material
