# Supplementary material for: Non-invasive methods for the determination of body and carcass composition in livestock: dual-energy X-ray absorptiometry, computed tomography, magnetic resonance imaging and ultrasound: invited review
Source: Animal. 2015 Mar 6;9(7):1250–64. doi: 10.1017/S1751731115000336 (PMC4492221; doi:10.1017/S1751731115000336)
Supplement: Supplementary file 1 [file S1751731115000336sup001.docx]

**Supplementary Material S1. References (before 2010)**

Andrássy-Baka G, Romvári R, Sütő Z, Szabó A, Horn P 2003. Comparative study of the body composition of different turkey genotypes by means of CT. Archiv Tierzucht 46, 285-293.

Baulain U and Henning M 2001. Untersuchungen zur Schlachtkörper- und Fleischqualität mit Hilfe von MR-Tomographie und MR-Spektroskopie. Archiv Tierzucht 44, 181-192.

Baulain U, Wiese M, Tholen E, Höreth R, Hoppenbrock KH 2003. Magnet-Resonanz-Tomographie als Referenztechnik zur Bestimmung der Körperzusammensetzung in der Leistungsprüfung beim Schwein. Mitteilungsblatt BAFF, der Bundesanstalt für Fleischforschung, Kulmbach 42(161), 251-257.

Bascom SS 2002. Jersey Calf Management, Mortality, and Body Composition. PhD thesis, Virginia Polytechnic State Institute and State University, USA.

Brandberg J 2009. Computed tomography and magnetic resonance imaging in determination of human body composition methodological and applied studies. PhD Thesis, Department of Radiology, Institute of Clinical Sciences, University of Gothenburg, Sweden

Casey JC, Miles CA 1974. Determination of the fat content of meat using nuclear magnetic resonance. Journal of the Science of Food and Agriculture 25, 1155-1161.

Collewet G, Bogner P, Allen P, Busk H, Dobrowolski A, Olsen E, Davenel A 2005. Determination of the lean meat percentage of pig carcasses using magnetic resonance imaging. Meat Science 70, 563–572.

Crabtree NJ, Leonard MB, Zemel BS 2007. Dual-energy X-ray absorptiometry. In: Current Clinical Practice: Bone Densitometry in Growing Patients: Guidelines for Clinical Practice (eds. AJ Sawyer, LK Bachrach and EB Fung), 41-57, Humana Press, Totowa, NJ, USA.

Davenel A, Seigneurin F, Collewet G, Rémignon H 2000. Estimation of poultry breast meat yield: magnetic resonance imaging as a tool to improve the positioning of ultrasonic scanners. Meat Science 56, 153-158.

Doeschl-Wilson AB, Green DM, Fisher AV, Carroll SM, Schofield CP, Whittemore CT 2005. The relationship between body dimensions of living pigs and their carcass composition. Meat Science 70, 229–240.

Drennan MJ, McGee M, Conroy SB, Keane MG, Kenny DA, Berry DP 2009. The relationship between various live animal scores/measurements and carcass classification for conformation and fatness with meat yield and distribution, and ultimate carcass value. Beef Production Series No. 89, 1-40.

Dunshea FR, Suster D, Eason PJ, Warner RD, Hopkins DL, Ponnampalam EN 2007. Dual energy X-ray absorptiometry (DXA) can be used to predict half-carcass composition in lambs. Australian Journal of Experimental Agriculture 47, 1165--1171.

Fantazzini P, Gombia M, Schembri P, Simoncini N, Virgili R 2009. Use of magnetic resonance imaging for monitoring Parma dry-cured ham processing. Meat Science 82, 219-227.

Font-i-Furnols M and Gispert M 2009. Comparison of different devices for predicting the lean meat percentage of pig carcasses. Meat Science 83, 443-446.

Giles LR, Eamens GJ, Arthur PF, Barchia IM, James KJ, Taylor RD 2009. Differential growth and development of pigs as assessed by X-ray computed tomography. Journal of Animal Science 87, 1648–1658.

Glasbey CA and Robinson CD 2002. Estimators of tissue proportions from X-ray CT images. Biometrics 58, 928-936.

Glasbey CA, Robinson CD, Young M 1999. Segmentation of X-ray CT images using stochastic templates. Proceedings 10th International Conference on Image Analysis and Processing, Venice, Italy, 746-751.

Gong QY, Phoenix J, Kemp GJ, García-Fiñana M, Frostick SP, Brodie DA, Edwards RH, Whitehouse GH, Roberts N 2000. Estimation of body composition in muscular dystrophy by MRI and stereology. Journal of Magnetic Resonance Imaging 12, 467-475.

Groeneveld E, Kallweit E, Henning M and Pfau A 1983. Evaluation of body composition of live animals by X-ray and NMR computed tomography. CEC Meeting: In Vivo Measurement of Body Composition in Meat Animals. Bristol, UK, November 30 - December 1, 84-89.

Hampe J, Nüske S, Scholz AM, Förster M 2005. Untersuchungen zur Körperzusam-mensetzung und zum Wachstum von Kälbern unterschiedlicher genetischer Herkunft mittels Dualenergie–Röntgenabsorptiometrie (DXA). Archiv Tierzucht 48, 428-444.

Hanna G and Cuschieri A 2001. Image display technology and image processing. World Journal of Surgery 25, 1419–1427.

Hogreve F 1938. Untersuchungen über die Fettbildung wachsender Mastschweine mittels der Röntgendurchleuchtung. Züchtungskunde 13, 178-191.

Holló G, Szücz E, Tözsér J, Holló I, Seregi J, Repa I 2008. The use of X-ray computer tomography for slaughter value estimation of cattle. Archiva Zootechnica 11, 57-64.

Hopkins DL, Ponnampalam EN, Warner RD 2008. Predicting the composition of lamb carcases using alternative fat and muscle depth measures. Meat Science 78, 400–405.

Hull H, He Q, Thornton J, Javed F, Wang J, Pierson RN, Gallagher D 2009. iDXA, Prodigy, and DPXL dual-energy X-ray absorptiometry whole-body scans: a cross-calibration study. Journal of Clinical Densitometry 12, 95–102.

Johansen J, Egelandsdal B, Røe M, Kvaal K, Aastveit AH 2007. Calibration models for lamb carcass composition analysis using computerized tomography (CT) imaging. Chemometrics and Intelligent Laboratory Systems 87, 303–311.

Johnson TRC 2009. Dual-energy CT – technical background. In: Multislice CT. 3rd Revised Edition. (eds. MF Reiser, CR Becker, K Nikolaou, G Glazer), 65-73, Springer, Germany.

Jones HE, Lewis RM, Young MJ, Simm G 2004. Genetic parameters for carcass composition and muscularity in sheep measured by X-ray computer tomography, ultrasound and dissection. Livestock Production Science 90, 167–179.

Jose CG, Pethick DW, Jacob RH, Gardner GE 2009. CT scanning carcases has no detrimental effect on the colour stability of M. longissimus dorsi from beef and sheep. Meat Science 81, 183-187.

Judas M, Höreth R, Dobrowolski A 2005. Computertomographie als Methode zur Analyse der Schlachtkörper von Schweinen. Mitteilungsblatt Fleischforschung Kulmbach 44, 145-151.

Junkuszew A and Ringdorfer F 2005. Computer tomography and ultrasound measurement as methods for the prediction of the body composition of lambs. Small Ruminant Research 56, 121-125.

Kalender WA 1988. Densitometrie mit Zwei-Spektren-Verfahren in der Computertomographie. In: Quo Vadis CT? (eds. C Claussen and R Felix), 312-324, Springer, Germany.

Kalender WA 2006. X-ray computed tomography. Physics in Medicine and Biology 51, R29–R43.

Kallweit E 1992. Measurement of body, carcass, and tissue composition in meat animals by non-invasive methods. Pig News and Information 13, 147N-148N.

Karamichou E, Richardson RI, Nute GR, McLean KA, Bishop SC 2006. Genetic analyses of carcass composition, as assessed by X-ray computer tomography, and meat quality traits in Scottish Blackface sheep. Animal Science 82, 151–162.

Kato H, Kuroda M, Yoshimura K, Yoshida A, Hanamoto K, Kawasaki S, Shibuya K, Kanazawa S 2005. Composition of MRI phantom equivalent to human tissues. Medical Physics 32, 3199 – 3208.

Kleczek K, Wawro K, Wilkiewicz-Wawro E, Makowski W, Konstantynowicz D 2009. Relationships between breast muscle thickness measured by ultrasonography and meatiness and fatness in broiler chickens. Archiv Tierzucht 52, 538-545.

Kolstad K. 2001. Fat deposition and distribution measured by computer tomography in three genetic groups of pigs. Livestock Production Science 67, 281–292.

Kolstad K, Mørkøre T, Thomassen MS 2008. Quantification of dry matter % and liquid leakage in Atlantic cod (Gadus morhua) using computerised X-ray tomography (CT). Aquaculture 275, 209-216.

Kongsro J, Røe M, Aastveit AH, Kvaal K, Egelandsdal B 2008. Virtual dissection of lamb carcasses using computer tomography (CT) and its correlation to manual dissection. Journal of Food Engineering 88, 86-93.

Kopp AF, Klingenbeck-Regn K, Heuschmid M, Küttner A, Ohnesorge B, Flohr T, Schaller S, Claussen CD 2000. Multislice Computed Tomography: Basic Principles and Clinical Applications. Electromedica 68, 94-105.

Kreuzer B 2008. Einfluss unterschiedlicher Energiegehalte in Alleinfuttermitteln der ökologischen Putenmast auf den Wachstumsverlauf eines Putengenotyps, gemessen mittels Dualenergie-Röntgenabsorptiometrie (DXA). PhD thesis, Veterinary Faculty, Ludwig-Maximilians University Munich, Germany.

Kronacher C and Hogreve F 1936. Beiträge zur Kenntnis der Grundlagen der Beckenformen bei verschiedenen Schweinerassen, gewonnen an Hand röntgenologischer Studien. Zeitschrift für Züchtung. Reihe B, Tierzüchtung und Züchtungsbiologie einschließlich Tierernährung 35, 161–167.

Kröger C, Bartle CM, West JG 2006. “Hybrid” calibrations of a dual energy X-ray scanner for material testing. Journal of Physics: Conference Series 41, 323–330.

Kullberg J, Angelhed JE, Lönn L, Brandberg J, Ahlström H, Frimmel H, Johansson L 2006. Whole-body T1 mapping improves the definition of adipose tissue: consequences for automated image analysis. Journal of Magnetic Resonance Imaging 24, 394–401.

Kullberg J, Ahlström H, Johansson L, Frimmel H 2007. Automated and reproducible segmentation of visceral and subcutaneous adipose tissue from abdominal MRI. International Journal of Obesity 31, 1806–1817.

Kvame T and Vangen O 2007. Selection for lean weight based on ultrasound and CT in a meat line of sheep. Livestock Science 106, 232-242.

Kvame T, Brenøe UT, Vangen O 2006. Body tissue development in lambs of two genetic lines analysed by X-ray computer tomography. Small Ruminant Research 65, 242-250.

Lambe NR, Navajas EA, McLean KA, Simm G, Bünger L 2007. Changes in carcass traits during growth in lambs of two contrasting breeds, measured using computer tomography. Livestock Science 107, 37-52.

Lambe NR, Navajas EA, Schofield CP, Fisher AV, Simm G, Roehe R, Bünger L 2008. The use of various live animal measurements to predict carcass and meat quality in two divergent lamb breeds. Meat Science 80, 1138-1149.

Laurent W, Bonny JM, Renou JP 2000. Muscle characterisation by NMR imaging and spectroscopic techniques. Food Chemistry 69, 419-426.

Latorre MA, Pomar C, Faucitano L, Gariépy C, Méthot S 2008. The relationship within and between production performance and meat quality characteristics in pigs from three different genetic lines. Livestock Science 115, 258–267

Locsmandi L, Romvari R, Bogenfürst F, Szabo A, Molnar M, Andrassy-Baka G, Horn P 2005: *In vivo* studies on goose liver development by means of computer tomography. Animal Research 54, 135–145.

Macfarlane JM, Lewis RM, Emmans GC, Young MJ, Simm G 2009. Predicting tissue distribution and partitioning in terminal sire sheep using x-ray computed tomography. Journal of Animal Science 87, 107-118.

MacNeil MD and Northcutt SL 2008. National cattle evaluation system for combined analysis of carcass characteristics and indicator traits recorded by using ultrasound in Angus cattle. Journal of Animal Science 86, 2518-2524.

Mandarim-De-Lacerda CA 2003. Stereological tools in biomedical research. Anais da Academia Brasileira de Ciências 75, 469-486.

Marcoux M, Faucitano L, Pomar C 2005. The accuracy of predicting carcass composition of three different pig genetic lines by dual-energy X-ray absorptiometry. Meat Science 70, 655–663.

Margeta V, Kralik G, Kušec G, Baulain U 2007. Lean and fat development in the whole body and hams of hybrid pigs studied by magnetic resonance tomography. Czech Journal of Animal Science 52, 130–137.

McEvoy FJ, Madsen MT, Strathe AB, Svalastoga E 2008. Hounsfield unit dynamics of adipose tissue and non-adipose soft tissues in growing pigs. Research in Veterinary Science 84, 300-304.

McEvoy FJ, Madsen MT, Nielsen MB, Svalastoga EL 2009. Computer tomographic investigation of subcutaneous adipose tissue as an indicator of body composition. Acta Veterinaria Scandinavica 51, 28.

Mercier J, Pomar C, Marcoux M, Goulet F, Thériault M, Castonguay FW 2006. The use of dual energy X-ray absorptiometry to estimate the dissected composition of lamb carcasses. Meat Science 73, 249-257.

Mitchell AD and Scholz AM 2009. Relationships among dual-energy X-ray absorptiometry (DXA), bioelectrical impedance (BIA), and ultrasound measurements of body composition of swine. Archiv Tierzucht 52, 28-39.

Mitchell AD, Conway JM, Scholz AM 1996. Incremental changes in total and regional body composition of growing pigs measured by dual-energy-x-ray absorptiometry. Growth Development and Aging 60, 113-123.

Mitchell AD, Scholz AM, Pursel VG 2000. Dual energy X-ray absorptiometry measurements of the body composition of pigs of 90- to 130-kilograms body weight. Annals of the New York Academy of Sciences 904, 85-93.

Mitchell AD, Scholz AM, Wang PC, Song H 2001. Body composition analysis of the pig by magnetic resonance imaging. Journal of Animal Science 79, 1800-1813.

Mitchell AD, Scholz AM, Pursel VG 2002. Prediction of the in vivo body composition of pigs based on cross-sectional region analysis of dual energy X-ray absorptiometry (DXA) scans. Archiv Tierzucht 45, 535-545.

Mitchell AD, Scholz AM, Pursel VG 2003. Prediction of pork carcass composition based on cross-sectional region analysis of dual energy X-ray absorptiometry (DXA) scans. Meat Science 63, 265-271.

Monziols M, Collewet G, Mariette F, Kouba M, Davenel A 2005. Muscle and fat quantification in MRI gradient echo images using a partial volume detection method. Application to the characterization of pig belly tissue. Magnetic Resonance Imaging 23, 745–755.

Monziols M, Collewet G, Bonneau M, Mariette F, Davenel A, Kouba M 2006. Quantification of muscle, subcutaneous fat and intermuscular fat in pig carcasses and cuts by magnetic resonance imaging. Meat Science 72, 146–154.

Müller S and Polten S 2004. Vergleichsuntersuchungen zur Ultraschall-Speckdickenmessung beim Schwein im Rahmen der Eigenleistungsprüfung. Archiv Tierzucht 47, 249-260.

Nanton DA, Vegusdal A, Bencze Rørå AM, Ruyter B, Baeverfjord G, Torstensen BE 2007. Muscle lipid storage pattern, composition, and adipocyte distribution in different parts of Atlantic salmon (Salmo salar) fed fish oil and vegetable oil. Aquaculture 265, 230-243.

Navajas EA, Lambe NR, McLean KA, Glasbey CA, Fisher AV, Charteris AJL, Bünger L, Simm G 2007. Accuracy of in vivo muscularity indices measured by computed tomography and their association with carcass quality in lambs. Meat Science 75, 533-542.

Nissen PM, Busk H, Oksama M, Seynaeve M, Gispert M, Walstra P, Hansson I, Olsen E 2006. The estimated accuracy of the EU reference dissection method for pig carcass classification. Meat Science 73, 22–28.

Pearce KL, Ferguson M, Gardner G, Smith N, Greef J, Pethick DW 2009. Dual X-ray absorptiometry accurately predicts carcass composition from live sheep and chemical composition of live and dead sheep. Meat Science 81, 285-293.

Plank LD 2005. Dual-energy X-ray absorptiometry and body composition. Current Opinion in Clinical Nutrition and Metabolic Care 8, 305–309.

Ponnampalam EN, Butler KL, Hopkins DL, Kerr MG, Dunshea FR, Warner RD 2008. Genotype and age effects on sheep meat production. 5. Lean meat and fat content in the carcasses of Australian sheep genotypes at 20-, 30- and 40-kg carcass weights. Australian Journal of Experimental Agriculture 48, 893-897.

Romvári R, Szabó A, Karpati J, Kovach G, Bazar G, Horn P 2005. Measurement of belly composition variability in pigs by *in vivo* computed tomographic scanning. Acta Veterinaria Hungarica 53, 153–162.

Romvári R, Dobrowolski A, Repa I, Allen P, Olsen E, Szabó A, Horn P 2006. Development of a computed tomographic calibration method for the determination of lean meat content in pig carcasses. Acta Veterinaria Hungarica 54, 1-10.

Rosset A, Spadola L, Ratib O 2004. OsiriX: An Open-Source Software for Navigating in Multidimensional DICOM Images. Journal of Digital Imaging 17, 205-216.

Scholz AM and Förster M 2006. Genauigkeit der Dualenergie-Röntgenabsorptiometrie zur Ermit-tlung der Körperzusammensetzung von Schweinen in vivo. Archiv Tierzucht 49, 462-476.

Scholz AM and Baulain U 2009. Methoden zur Bestimmung der Körperzusammensetzung am lebenden Nutztier. Züchtungskunde 81, 86-96.

Scholz A, Baulain U, Kallweit E 1993. Quantitative Analyse von Schnittbildern lebender Schweine aus der Magnet-Resonanz-Tomographie. Züchtungskunde 65, 206-215.

Scholz A, Soffner P, Littmann E, Peschke W, Förster M 2002. Genauigkeit der Dualenergie-Röntgenabsorptiometrie (DXA) zur Ermittlung der Schlachtkörperzusammensetzung von Schweinehälften (kalt, 30-39 kg) anhand der EU-Referenzzerlegung. Züchtungskunde 74, 376-391.

Scholz AM, Nüske S, Förster M 2003. Body composition and bone mineralization in calves of different genetic origin by using dual energy X-ray absorptiometry. Acta Diabetologica 40 (Suppl.1), S91–S94.

Scholz AM, Mitchell AD, Förster M, Pursel VG 2007. Two-site evaluation of the relation between *in vivo* and carcass dual energy x-ray absorptiometry (DXA) in pigs. Livestock Science 110, 1–11.

Schöllhorn B and Scholz AM 2007. Untersuchungen zur Anwendbarkeit der Dualenergie-Röntgenabsorptiometrie (DXA) für die Messung der Ganzkörperzusammensetzung bei zwei Putengenotypen. Archiv für Geflügelkunde 71, 228–236.

Schreiweis MA, Orban JI, Ledur MC, Moody DE, Hester PY 2005. Validation of dual-energy X-ray absorptiometry in live White Leghorns. Poultry Science 84, 91–99.

Schröder UJ and Staufenbiel R 2006. Invited review: methods to determine body fat reserves in the dairy cow with special regard to ultrasonographic measurement of backfat thickness. Journal of Dairy Science 89, 1–14.

Skjervold H, Grønseth K, Vangen O, Evensen A 1981. In vivo estimation of body composition by computerized tomography. Zeitschrift für Tierzüchtung und Züchtungsbiologie 98, 77-79.

Starck JM, Dietz MW and Piersma T 2001. The assessment of body composition and other parameters by ultrasound scanning. In: Body Composition Analysis of Animals (ed. JR Speakman), 188-210, Cambridge University Press, New York, USA

Stouffer JR 2004. History of ultrasound in animal science. Journal of Ultrasound in Medicine 23, 577–584.

Suster D, Leury BJ, Hofmeyr CD, D’Souza DN, Dunshea FR 2004. The accuracy of dual energy X-ray absorptiometry (DXA) weight, and P2 back fat to predict half-carcass and primal-cut composition in pigs within and across research experiments. Australian Journal of Agricultural Research 55, 973-982.

Suther S 2009. Genetics and marbling. ANGUS Journal September, 112-113.

Swennen Q, Janssens GPJ, Geers R, Decuypere E, Buyse J 2004. Validation of dual-energy X-ray absorptiometry for determining in vivo body composition of chickens. Poultry Science 83,1348–1357.

Szabó C and Babinszky L 2008. The effect of carcass temperature and treatment on the computer tomography based tissue separation. Acta agriculturae Slovenica, S 2, 181–185.

Szabó C and Babinszky L 2009. Minimum slicing interval and frequency for CT-based prediction of pig’s body composition. Italian Journal of Animal Science 8 (Suppl. 3), 246-248.

Temple RS, Stonaker HH, Howry D, Posakony G, Hazaleus HM 1956. Ultrasonic and conductivity methods for estimating fat thickness in live cattle. Proceedings Western Sec-tion American Society of Animal Production, July 15-18, Reno, Nevada, USA, 7, 477–481.

Tholen E, Baulain U, Henning MD, Schellander K 2003. Comparison of different methods to assess the composition of pig bellies in progeny testing. Journal of Animal Science 81, 1177-1184.

Thomas AMK, Banerjee AK, Busch U (eds.) 2005. Classic papers in modern diagnostic radiology. Springer-Verlag, Berlin Heidelberg, Germany.

Török M, Polgar JP, Kocsi G, Farkas V, Szabo F 2009. Correlation of ultrasonic measured ribeye area and fat thickness to the certain traits measured on slaughtered bulls. Archiv Tierzucht 52, 23-27.

Ulzheimer S and Flohr T 2009. Multislice CT: Current technology and future developments. In: Multislice CT, 3^rd^ revised edition, (eds. MF Reiser, CR Becker, K Nikolaou and G Glazer) Medical Radiology, 3-23, Springer, Berlin Heidelberg, Germany.

Vester-Christensen M, Erbou SGH, Hansen MF, Olsen EV, Christensen LB, Hviid M, Ersbøll BK, Larsen R 2009. Virtual dissection of pig carcasses. Meat Science 81, 699–704.

Visscher PM, Hill WG, Wray NR 2008. Heritability in the genomics era - concepts and misconceptions. Nature Reviews Genetics 9, 255-266.

Vogt FM, Ruehm S, Hunold P, de Greiff A, Nuefer M, Barkhausen J, Ladd SC 2007. Schnelle Ganzkörperfettmessung mittels MRT: Quantifizierung und Topografie. Fortschritte auf dem Gebiet der Röntgenstrahlen und der bildgebenden Verfahren 179, 480–486.

Vogt C, Laihem K, Wiebusch C 2008. Speed of sound in bubble-free ice. The Journal of the Acoustical Society of America 124, 6 pgs.

von Korn S, Baulain U, Arnold M, Brade W 2005. Nutzung von Magnet-Resonanz-Tomographie und Ultraschalltechnik zur Bestimmung des Schlachtkörperwertes beim Schaf. Züchtungskunde 77, 382 – 393.

Wolf BT and Jones DA 2007. Inheritance of an *in vivo* leg conformation score in Texel lambs and its association with growth, ultrasonic measurements and muscularity. Livestock Science 110, 133–140.

Wood SE 2004. The effectiveness of dual energy X-ray absorptiometry to non-invasively determine body composition of hybrid striped bass. Master Thesis, University of Maryland, College Park, MD, USA.

Young MJ, Simm G, Glasbey CA 2001. Computerised tomography for carcass analysis. Proceedings of the British Society of Animal Science, 250-254.

Youssao IAK, Verleyen V, Leroy PL 2002. Prediction of carcass lean content by real-time ultrasound in Pietrain and negative stress Pietrain. Animal Science 75, 25-32.
